# A Rapid and International Applicable Diagnostic Device for Cobra (Genus *Naja*) Snakebites

**DOI:** 10.3390/toxins12090572

**Published:** 2020-09-05

**Authors:** Jing-Hua Lin, Wang-Chou Sung, Jiunn-Wang Liao, Dong-Zong Hung

**Affiliations:** 1 Graduate Institute of Veterinary Pathobiology, National Chung Hsing University, Taichung 40227, Taiwan; jh.cooltm@gmail.com; 2 National Institute of Infectious Diseases and Vaccinology, National Health Research Institutes, Miaoli 35053, Taiwan; sung23@nhri.edu.tw; 3 Division of Toxicology, China Medical University Hospital, Taichung 40447, Taiwan

**Keywords:** ICT-Cobra, cobra, snakebite, venom detection, transregional, diagnostic tool

## Abstract

Cobra snakes (genus *Naja*) are some of the most dangerous snake species in Asia and Africa, as their bites cause severe life-threatening respiratory failure and local tissue destruction, especially in the case of late diagnosis. The differential diagnosis of snakebite envenomation still mainly relies upon symptomatology, the patient’s description, and the experience of physicians. We have designed a rapid test, immunochromatographic test of cobra (ICT-Cobra), which obtained fair results in improving the diagnosis and treatment of *Naja (N.) atra* snakebites in Taiwan. In this study, we further investigated the feasibility of applying the kit for the detection of other cobra venoms based on the potential interspecies similarity. We firstly demonstrated the cross-reactivity between eight venoms of medically important cobra species and the rabbit anti-*N. atra* IgG that was used in ICT-Cobra by Western blotting and sandwich enzyme-linked immunosorbent assay. Then, ICT-Cobra was used to detect various concentrations of the eight venoms to elucidate its performance. Noticeable correlations between the cross-reactivity of venoms from genus *Naja* snakes and existing geographical characteristics were found. ICT-Cobra could detect venoms from other Asian cobras with variable detection limits comparable to those observed for *N. atra*, but the kit was less successful in the detection of venom from African cobras. The similar but slightly different venom components and the interaction between venom and rabbit anti-*N. atra* IgG led to variations in the detection limits. The transcontinental usage of ICT-Cobra might be possible due to the cross-reactivity of antibodies and similarities among the larger-sized proteins. This study showed that the close immunological relationships in the genus *Naja* could be used to develop a venom detection kit for the diagnosis of cobra envenomation in both Asian and African regions. Additional clinical studies and technical adjustments are still needed to improve the efficacy and broadening the application of ICT-Cobra in the future.

## 1. Introduction

Snake envenomation has always been a global health issue, especially in rural areas and areas with inadequate medical service in tropical and subtropical countries [1,2]. The World Health Organization (WHO) categorized snakebites as a high-priority neglected tropical disease (NTD) in 2018 and declared a global goal to “end the epidemics” by 2030 [3,4,5,6]. In the geographical hotspots for snakebites, such as South Asia, South-East Asia, and sub-Saharan Africa, the cobra (genus *Naja* (*N.*)) bite is one of the most common and causes of severe envenomation. Envenomation is characterized by local necrosis, neurological paralysis, and chronic musculoskeletal disability [2,7,8]. These devastating injuries still challenge all healthcare providers [4,9]. Although the cobra snake behaves as characteristically rearing up and spreading a hood, which is accompanied by hissing in defense, its bite has been frequently and unwittingly mistaken for that of other snakes [7,9,10,11]. Swelling, ecchymosis, and local tissue necrosis are the common initial clinical manifestations of cobra and viper bites. The administration of the correct and specific antivenom, if confusion occurs at all, is typically delayed. Therefore, snake species determination is of primary importance to select the appropriate antivenom and to obtain better outcomes [9]. Inadequate dose or inappropriate specificity of antivenom have been identified as a contributing factor of fatal outcome [2]. Efficacious and user-friendly rapid tests might be indispensable in determining the correct diagnosis if the offending snake is not captured or is misidentified. Sensitive, specific, and rapid diagnostic testing not only paves the way for effective snakebite treatment, but also plays a critical role in preventing detrimental effects or complications in patients of cobra bite [2,6,12].

For decades, there have been numerous serological methods used for snake bite diagnosis. Due to the sensitivity, enzyme-linked immunosorbent assay (ELISA) has long been established as a useful tool for qualitative and quantitative detection of snake venom in biological fluids [9,10,13,14]. However, the requirement for special equipment and reagents as well as the time-consuming nature of the assay is a major obstacle for its use in impoverished areas where populations usually suffer from a higher incidence of snakebites [15,16,17]. Lateral flow assay (LFA), another common immunological diagnostic tool, might be suitable for overcoming the above disadvantages [18,19]. Based on LFA, we developed a workable rapid test, immunochromatographic test of cobra (ICT-Cobra), to diagnose and distinguish cobra (*N. atra*) bites from five krait or viper snake bites in Taiwan with low detection limit (5 ng/mL). The strips, based on the ELISA results, had 83.3% sensitivity and 100% specificity, and showed a strong agreement between these two diagnostic tools [20]. The ICT-Cobra helps physicians confirm the cobra bite quickly, which means that the victims are subjected to fewer surgical interventions and have a better prognosis due to early antivenom administration.

Since the 19th century, antivenom has been the only available antidote for snake envenomation therapy. However, there are over two hundred venomous snakes of medical importance in the world. It would be a huge task or even impossible to develop a universal drug or antivenom for treatment of most venomous snake bites around the world. To solve this problem and collaborate with the WHO to find a better solution for responding to neglected tropical public health issues, many studies have investigated this issue and have discovered antivenoms with the potential capability for cross-neutralization of venoms from cross-regional or pan-species of snakes [21,22,23,24]. For example, the venom of *N. kaouthia* was the only *Naja* genus venom used to generate neuro-polyvalent snake antivenom (NPAV). However, this antivenom can effectively neutralize heterologous venoms, such as *N. sumatrana*, *N. siamensis*, and *N. sputatrix* venoms, although it is less effective against *N. naja*, *N. melanoleuca*, and *N. nigricollis* venoms and has only a weak or even a negative effect on the highly lethal venoms of *N. haje* and *N. philippinensis* [21]. Another commercial *Bothrops* antivenom (CAv), which was generated from a mixture of venoms (*Bothrops (B.) alternatus*, *B. jararaca*, *B. neuwiedi*, *B. jararacussu*, and *B. moojeni*), was suggested to treat *B. fonsecai* envenomation in Brazil [24]. The cross-neutralization by some specific antivenoms of heterologous snake venoms was discussed due to the similarity of the composition of the characteristic venom proteins. The similarities in the protein composition led to the development of a potential strategy to produce broad-spectrum antivenom against pan-Asian Elapidae snake bites by using a mixture of crude venoms and toxin fractions [25]. Most results were promising and showed that the immunological cross-reaction between the antibody and the complex venom proteins of different species might be useful in overcoming the medical issues caused by the great diversity of snake venoms. Similarly, we thought that immunological cross-reactivity might also be exploited to create a transregional diagnostic device.

Thus, the aim of this study was to test the principle and the possibility of expanding the scope of ICT-Cobra for rapid diagnosis of *Naja* genus snakebites in Asia, or even in Africa. The diversity or geographical variation of venom components or contents, even within a single species, has been frequently mentioned. However, some of the similarities among cobra species in terms of the immunological cross-reactivity of their venom with antibodies might enable the applicability of ICT-Cobra in these areas.

## 2. Results

### 2.1. Cross-Reactivity between Rabbit Anti-N. atra IgG and Other Cobra Venoms

**Figure 1:**
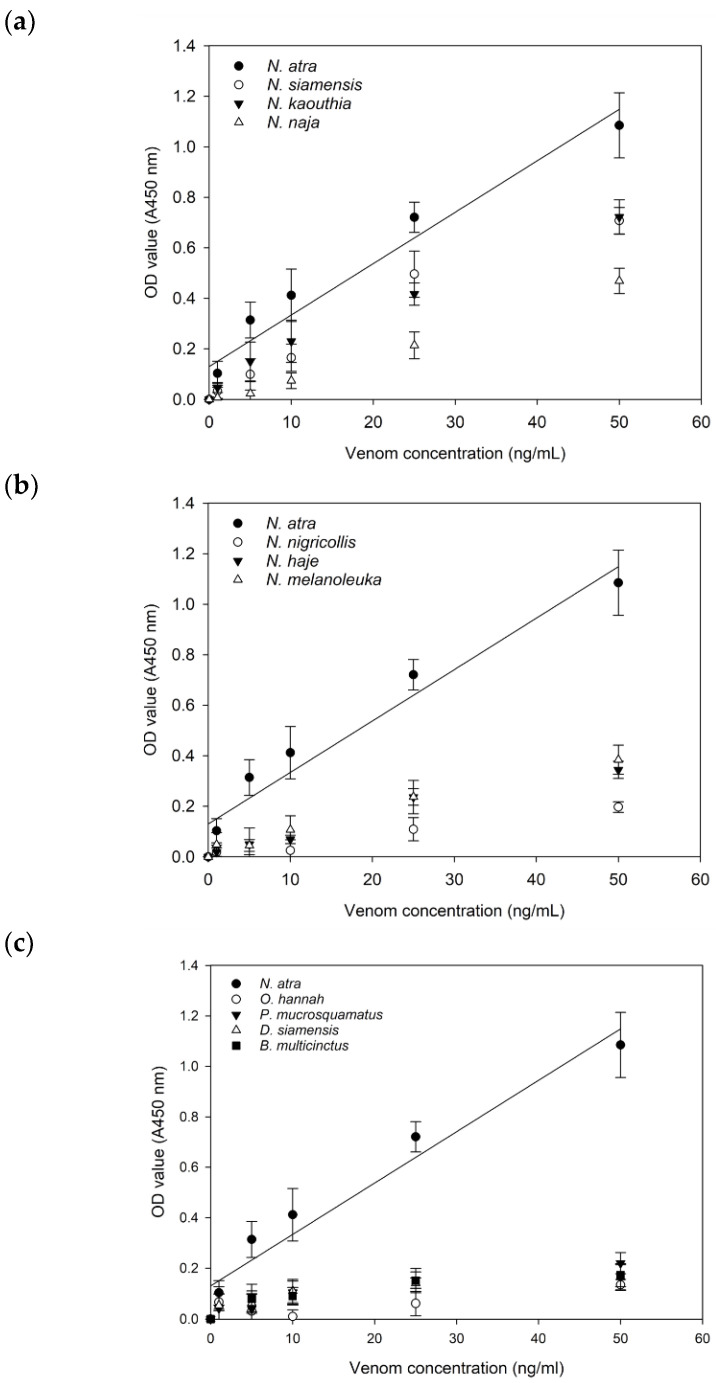
The cross-reactivity was assessed by sandwich ELISA. In all the above experiments, rabbit anti-*N. atra* IgG was used as a capture antibody and reacted with (**a**) Asian and (**b**) African cobra, as well as (**c**) other krait or viper venoms at concentrations of 0, 1, 5, 10, 25 and 50 ng/mL individually. The R^2^ value of the *N. atra* venom trend line is 0.947. All the OD values shown in figures were the results after background deduction and presented as the average of triplicate data points as mean ± SD.

Previously, we developed a sandwich ELISA method to diagnose *N. atra* snakebites in Taiwan and verify the clinical efficacy of ICT-Cobra [10,20]. Here, we applied this method and coated the plates with rabbit anti-*N. atra* IgG to study the possible immunological cross-reactivity between this antibody and heterogeneous cobra venoms from Asia or Africa. As shown in Figure 1, the dose-dependent trend showed good relevance between the venom of *N. atra* and the venom of three other Asian cobra species, namely, *N. siamensis*, *N. kaouthia*, and *N. naja* (Figure 1a), but not with the venom of three African cobra species, namely, *N. nigricollis*, *N. haje*, and *N. melanoleuca*. The absorbances of African cobra venoms recognized by anti-*N. atra* IgG were all below 0.4, even at a venom concentration of 50 ng/mL (Figure 1b). The results indicated that the similarities of snake venom do indeed show geographical differences. In other words, the binding capacity of rabbit anti-*N. atra* IgG for other Asian cobra venoms was comparable to that for *N. atra* venom but was weaker for venom from African cobras. Nearly no reactivity was observed between the rabbit anti-*N. atra* IgG and the venom of *Ophiophagus* (*O.*) *hannah*, *Protobothrops* (*P.*) *mucrosquamatus*, *Daboia* (*D.*) *siamensis*, and *Bungarus* (*B.*) *multicinctus* (Figure 1c).

### 2.2. The Protein Electrophoretic Profile of Cobra Venoms and Recognized by Rabbit Anti-N. atra IgG

**Figure 2:**
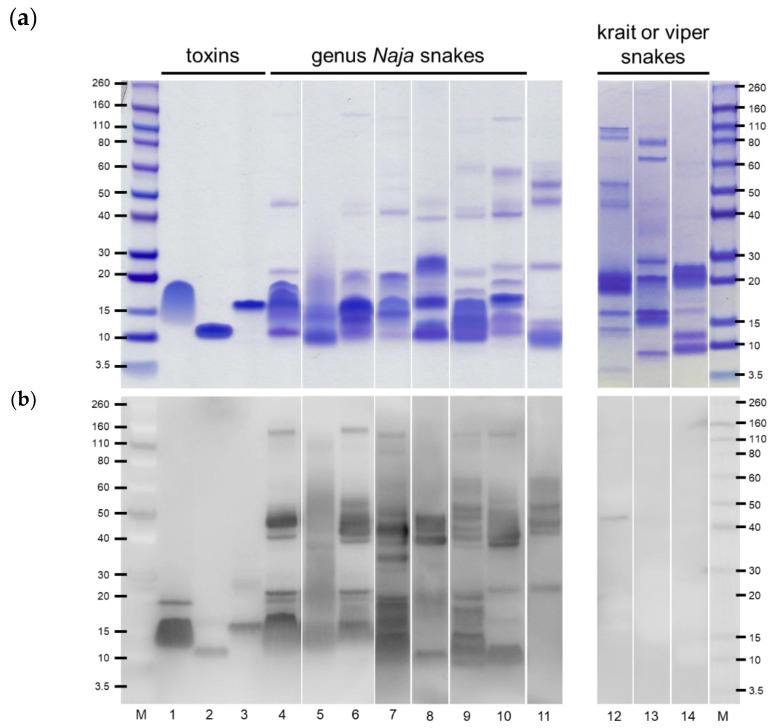
Gel electrophoresis profile of venom proteins and rabbit anti-*N. atra* IgG recognition. (**a**) Crude venoms from Asian and African cobras and isolated toxins were electrophoresed under non-reducing conditions and stained with Coomassie Brilliant Blue G-250. (**b**) The separated proteins were detected by rabbit anti-*N. atra* IgG by using Western blotting. Lane M: molecular weight markers; 1–3: cardiotoxin A3 (CTX A3), short-chain neurotoxin (sNTX), and phospholipase A_2_ (PLA_2_) toxin; 4: *N. atra*; 5: *N. siamensis*; 6: *N. kaouthia*; 7: *N. naja*; 8: *N. nigricollis*; 9: *N. haje*; 10: *N. melanoleuca*; 11: *O. hannah*; 12: *P. mucrosquamatus*; 13: *D. siamensis*; 14: *B. multicinctus*.

To study the immunological cross-reactivity, cobra venoms were separated by gel electrophoresis and probed by Western blotting. The isolated toxins, cardiotoxin A3 (CTX A3), short-chain neurotoxin (sNTX), and phospholipase A_2_ (PLA_2_), were subjected to gel electrophoresis at the same time as the benchmark low molecular weight toxins (Figure 2). The results showed that all cobra venoms from both Asian and African species had a similar protein profile, and their major components were low molecular weight proteins ranging from 10 to 20 kDa in size (Figure 2a). This indicated that CTX A3, sNTX, and PLA_2_ might play a major role in the abundances of low molecular weight proteins. Although the proteins with molecular weights larger than 30 kDa were relatively less abundant in venoms (Figure 2a), they were intensely recognized by rabbit anti-*N. atra* IgG, which led to the assumption that antigens with large molecules might induce the adaptive immune response of the host more readily (Figure 2b). Fewer low molecular weight proteins (<30 kDa) were recognized by the rabbit anti-*N. atra* IgG in African cobra venoms than in Asian cobra venoms. Despite the diversity in the intensity, unmistakable similarities and cross-reactivity were observed between most of the cobra venoms and the rabbit polyclonal immunoglobulin that was raised and produced only in response to *N. atra* venom. To assess the geographic variation on these regional venoms, HPLC was introduced to compare their protein profile, and the acquired chromatograms showed that a high proportion of elution peaks was overlapped between the Asian cobra venoms. In contrast, the major elution peaks derived from *N. atra* venom was not observed in the chromatogram of African cobra venoms, suggesting a significant variance in either venom components or protein properties (Figure S1). For the king cobra venom, it is interesting that we observed only a slight reaction to rabbit anti-*N. atra* IgG by the 20–60 kDa proteins, but there was no probe signal from proteins with molecular weights below 20 kDa (Figure 2b). The nearly absent antibody probe signals were observed in venoms of krait and viper snakes even though the proteins were strongly stained (Figure 2a,b).

### 2.3. ICT-Cobra for Transregional Cobra Venom Detection In Vitro

**Figure 3:**
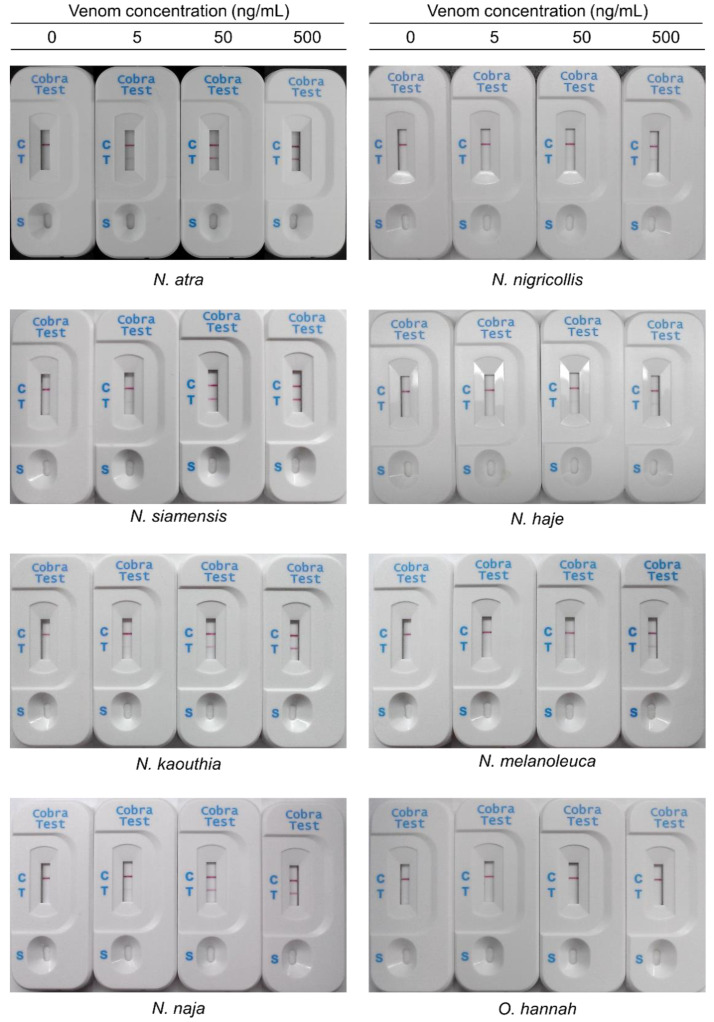
Examples of the use of immunochromatographic test of cobra (ICT-Cobra) to detect eight representative cobra venoms in vitro. Venom samples were freshly dissolved in fetal bovine serum (FBS) at concentrations of 0, 5, 50, and 500 ng/mL, and 90 µL of solution was loaded onto the sample zone of ICT-Cobra. These pictures were taken 20 min later. The detection limit of Asian cobras was noted to be 5 or 10 ng/mL, nonetheless, the detection limit of African cobras was up to be 50 or 500 ng/mL. The labels on the plastic cassette: C, control line, checking the immunochromatographic function; T, test line, detecting the presence of venom in the sample; S, sample loading zone.

Based on the similarities of the protein profiles and immunological cross-reactivity among the venoms from several *Naja* genera, ICT-Cobra was used to test these medically important cobra venoms from Asia and Africa to determine the detection limit in vitro. The results showed that the detection limit was low (5 ng/mL) for most Asian cobra venoms, except for the venom of *N. siamensis* (10 ng/mL). ICT-Cobra could also detect some African cobra venoms with a detection limit of up to 50 (*N. haje*), 100 (*N. melanoleuca*), or 500 ng/mL (*N. nigricollis*) (Figure 3, Table 1). By venoms spiked into healthy human serum, ICT-Cobra could still detect lower venom concentration of Asian cobra than African cobra venoms and would not produce the cross-reactivity with venoms of king cobra, krait, and viper snakes (Figure S2). This means that, based on the intra-genus similarity, ICT-Cobra has the potential to be used in Asian and African regions but could not be used for the diagnosis of king cobra bites.

## 3. Discussion

For many years, the WHO has collaborated with scientists and public health staff globally to find a solution to the problem of snakebite envenomation. Despite many challenges, with a strong mandate, the WHO has declared its intention to take action on a globally coordinated response strategy for snakebites [26]. Nothing is more important than developing a comprehensive, efficacious, affordable, stable, and cross-regional diagnosis tool and medicine for successful snakebite management. ICT-Cobra may serve in such a capacity; moreover, it could transcend territorial restrictions. The present study illustrated that the principles of venomic similarities and antivenomic cross-reactivity might be applied to develop a generic diagnosis kit for snakebites on several continents by using the cobra as an example (Figure 1). The similarities of the protein profiles of Asian and African *Naja* species venoms might be the reason for this.

It is well established that three-finger toxins and PLA_2_ account for 64–95.2% of *Naja* snake venom proteins [22,27,28,29,30,31,32,33]. Our gel electrophoresis results were in line with other earlier reports and showed that CTX A3, sNTX, and PLA_2_ comprise almost all of the major components of Asian and African cobra venoms (Figure 2a). The other obvious protein bands, which were less abundant but had larger molecular weights, might represent SVMP, cysteine-rich secretory protein (CRISP), cobra venom factor (CVF), and L-amino acid oxidase (LAAO). These are usually regarded as toxicity-enhancing factors or physiological function inhibitors of prey [34,35,36,37]. Our results indicated that the protein profiles of the seven genera of *Naja* snakes were similar but that the protein profile of the king cobra was different (Figure 2a). Although the RP-HPLC results revealed the components among cobra venoms were diverse, the alignment of elution peaks with some overlapping might have a high chance that similar toxin or protein families exist (Figure S1). It needs more studies to reveal the exact proteins that are related to the performance of ICT-Cobra. Furthermore, most of the venom proteins, especially those of large molecular weight among these species could also be recognized by rabbit anti-*N. atra* IgG (Figure 2b). This might be the reason why ICT-Cobra could detect Asian or African cobra venoms with varied detection limits.

The degree of the similarity of the venom components and immunological cross-reactivity with rabbit anti-*N. atra* IgG showed some differences between these venoms. We found that the OD values of ELISA at a venom concentration of 25 ng/mL could be ranked in the following order: *N. siamensis* > *N. kaouthia* > *N. naja* (Figure 1). However, a question remains as to whether this ranking directly reflects the detection limit of ICT-Cobra. In sandwich ELISA, we used both rabbit and horse anti-*N. atra* antibodies as the capture reagents, but only rabbit anti-*N. atra* IgG was used in ICT-Cobra. Due to variations in antigen presentation by different animals, antivenom that was raised in different hosts might result in differences in the detection by ELISA and ICT-Cobra (Figure 1, Table 1). ICT-Cobra uses a high-quality antibody raised only from rabbits and achieved great performance for cross-continental *Naja* snake venom detection in vitro. It could detect the venoms of *N. kaouthia* and *N. naja* as well as *N. atra* (Table 1). The high degree of the immunological cross-reactivity of rabbit anti-*N. atra* IgG might play an important role in the detection ability of ICT-Cobra (Figure 2b). This result was also consistent with those of the proteomic studies in which great similarity was found between the venom proteins of *N. atra* and *N. kaouthia* [22,26]. It was a little disappointing that the detection ability of ICT-Cobra was found to be lower for *N. siamensis* venom, although the OD value was as high as that observed for *N. kaouthia* venom by ELISA; the two venoms showed a close relationship in venomic studies [22].

Not surprisingly, ICT-Cobra did not perform well in detecting African cobra venoms, with detection limits ranging from 50 ng/mL to 500 ng/mL. This meant that there were significant differences in the venom compositions and poor immunological cross-reactivity between rabbit anti-*N. atra* IgG and African cobra venoms (Figure S1 and Figure 2b). Furthermore, despite being recognized by the rabbit antibody, as shown in Figure 2b, CTX A3, sNTX, and PLA_2_, which are well-known and abundant cobra venom proteins, were poorly detected by ICT-Cobra (data not shown) because of their poor immunogenicity in antivenom production [30,38]. The possibility for the transcontinental usage of ICT-Cobra might mainly result from the similarity and cross-reactivity between the antibody and larger-sized proteins, such as proteins from SVMP, LAAO, and CRISP toxin families, which are found ubiquitously in Elapidae snake venoms. Additional proteomic information and clinical trials are needed to study the impact or efficacy of applying ICT-Cobra or developing a new kit for venom detection on the more than 20 species of the *Naja* genus.

The success of antivenom therapy and the achievement of good outcomes for cases of venomous snake bites usually depend on the earlier and correct diagnosis of the snake species involved [14,39]. Currently, several snakebite diagnostic tools have been developed and published. One of the few commercial products is the Snake Venom Detection Kit (SVDK), which is manufactured by the Seqirus and is known in use in the Australian continent and Papua New Guinea. SVDK was based on the ELISA technique and was developed for correctly identifying the corresponding antivenom produced by the same company [40]. Because of its convenience and usability, LFA is another actively adopted diagnostic tool that has been used in the past few years. The quality of the specific antibodies used in LFA usually plays the most important role in the sensitivity and specificity of the devices. Monoclonal antibodies have been used for the capture and coating in the same platform for epitopes of a single antigen in many LFA studies [41,42,43]. However, creating a pool of monoclonal antibodies against all toxin families do not work for snake venoms due to their complex protein composition. Some studies have designed snake envenomation detection strips by using a species-specific antibody (SSAb), which is an ultra-specific antibody, to improve its sensitivity and specificity [44,45]. However, multistep purification would not only cause the loss of more specific antibodies but also increase the cost of the device. Moreover, this undoubtedly limits the scope of the application and the possibility of transregional use. The results of this study revealed that the similarity of the venom protein and cross-reactivity profiles suggest that ICT-Cobra or other similar devices in the future could be used more widely in Asian and African regions. In the light of our experiences of immunization, every 10 mL of rabbit serum could extract 18.43 mg of specific antibodies that is sufficient to produce about 1000 ICT-Cobra tests. The one-step affinity purification achieved a balance between the proper isolation of specific antibody and the cost of kit production at the same time. It was reported that the concentration of most snake venoms in serum was above 10 ng/mL soon after being envenomed, causing clinical symptoms. In some patients, the venom concentration could exceed 100 ng/mL, and even higher to 1000 ng/mL [10,46]. Thus, when using ICT-Cobra, in terms of the detection limit, 5–10 ng/mL is sufficient for Asian cobra envenomation assessment. For African cobra snakebites, the application would be limited and needs more investigation and optimization in the future. However, 50 ng/mL might be enough to aid clinical diagnosis and antivenom treatment in the case of significant envenomation to prevent severe disability and complications resulting from an incorrect and/or delayed diagnosis [46].

ICT-Cobra had previously been used in a small-scale clinical test in Bangladesh. Mortality and morbidity due to venomous snake bites are a great public health burden in Bangladesh, especially in rural areas. Bites from cobra snakes, including *N. kaouthia* and *N. naja*, are one of the leading causes of venomous snake envenomation in Bangladesh [47,48]. Syndromic approaches, not ELISA, are the main diagnostic methods due to the abundance of venomous snakes, including pit vipers, cobras, and kraits, which are variably distributed in Bangladesh [47,48]. The cases of descending paralysis accompanied by severe local tissue injuries were suggested to be *Naja* spp. envenomation. At the request of physicians from Bangladesh, we donated some ICT-Cobra devices and performed a small-scale test at Chittagong Medical College Hospital, an important tertiary hospital in Bangladesh. From 2012 to 2013, a total of 35 patients with cobra bites that were diagnosed by clinical histories and specimens brought with patients were tested. A positive result in the serum was found in 19 patients, and the detection rate of ICT-Cobra for *N. kaouthia* or *N. naja* venom was supposed to be 54.3% [49], which is not as good as that observed in in vitro studies. This disparity could be explained by the differences in sample conditions between clinical and in vitro tests. We are also obligated to raise or produce antibody of higher quality and perform more clinical investigations; and the efficacy of the rapid test kit applied in this area should be proved synchronously by ELISA or other confirmed tests in the future. Moreover, patients in Bangladesh customarily apply firm ligatures proximal to the bite site, and this might result in an abnormally high rate of symptoms-free envenoming and reduce the venom concentration in serum that can be detected by the kit [50]. Considering the different clinical approaches used in every area, unified and objective criteria for diagnosis, such as ICT-Cobra, should be used for further transregional clinical trials.

ICT-Cobra is not yet perfect and needs to be optimized more. First, the data shown in the study were mainly qualitative evidence; nonetheless, venom quantification and identification might further help for revealing the differences in origin. Second, in contrast to the commercial venoms used in this study, the wild specimens and their existence in blood might behave in different manners and need more investigation. Third, rabbit anti-*N. atra* IgG is a polyclonal antibody, and its quality is easily affected by individual variations. How to maintain the quality is important for ICT-Cobra production. Fourth, the ICT-Cobra had only been tested in Taiwan and Bangladesh and on limited cases of snakebite. More clinical trials and more validations in Asian and African territories are needed in the future. Fifth, the affinity-purified rabbit anti-*N. atra* IgG seemed not to be well competent as a pan-species antibody. It poorly recognized important small molecule toxins such as CTX A3, sNTX, and PLA_2_. Some investigators have tried to synthesize a recombinant consensus toxin or generate a novel toxin isomer to develop a broad-spectrum antivenom or antibody [26,51]. Such toxins could be used or combined with some crude venoms to generate a more sensitive and pan-specific antibody for optimizing immunochromatographic kits to improve future applications.

## 4. Conclusions

This is the first study of a lateral flow device that could be applied for cross-regional use based on the similarity and immunological cross-reactivity of cobras. The efficacy of the device was preliminarily proven. ICT-Cobra is an economic, practical, and transregional product for the identification of the most medically important cobra venoms in Asia and potentially in Africa. It might be applicable to distinguish the cobra and non-cobra snakebites. Additional collaborative field investigations and quality improvement are needed to prove the feasibility of its use in these areas.

## 5. Materials and Methods

### 5.1. Venoms and Toxins

Lyophilized snake venoms of *N. atra*, *N. kaouthia*, *N. naja*, *N. siamensis*, *N. melanoleuca*, *N. nigricollis*, and *O. hannah* (also known as the king cobra) were purchased from Latoxan (Portes-lès-Valence, France). These venoms were milked from South-East Asian (e.g., Taiwan, Thailand, and India), Cameroonian, and West and South African cobras, according to the product information. Another African cobra venom (*N. haje*) was purchased from the Kentucky Reptile Zoo, USA (Table 2). The CTX A3, sNTX and PLA_2_ were isolated from Taiwan cobra (*N. atra*) venom following the methods described in a previous study [52].

### 5.2. Antibodies and Reagents

Both rabbit and horse polyclonal antibodies raised against the venom of *N. atra* were donated by the Centers for Disease Control, Taiwan. These antivenoms were further affinity-purified by LTK BioLaboratories Co., Ltd. with an antigen-immobilized column system and dialyzed with PBS. Peroxidase-conjugated goat anti-rabbit IgG (H + L) and goat anti-horse IgG (H + L) were purchased from Jackson ImmunoResearch Inc. (West Grove, PA, USA). SureBlue Reserve™ TMB Microwell Peroxidase Substrate was purchased from SeraCare Life Sciences Inc. (Milford, MA, USA). Bolt™ 4–12% Bis-Tris Plus Gels and Novex^®^ Sharp Pre-stained Protein Standard were purchased from Thermo Fisher Scientific Inc. (Waltham, MA, USA). Immobilon-PSQ PVDF membrane and the chemiluminescent HRP substrate used in Western blotting were obtained from Merck Millipore (Burlington, MA, USA). Flat-bottom microtitration plates were purchased from Corning Inc. (Corning, NY, USA). All other chemicals were of analytical grade and were purchased from Sigma-Aldrich (St. Louis, MO, USA).

### 5.3. Sandwich Enzyme-Linked Immunosorbent Assay (Sandwich ELISA)

The procedure used for sandwich ELISA was based on our previously published method for venom detection in clinical samples, with modifications [10]. Microplates were coated with rabbit anti-*N. atra* IgG (20 µg/mL in carbonate buffer) and incubated overnight at 4 °C. We discarded the supernatant and added 150 µL PBST (0.01% Tween-20 in PBS) into each well five times to wash away the excess residue coating solution. Subsequently, 100 µL PBST containing 1% BSA was added, and the plate was incubated for 1 h to block the remaining binding sites in the wells. The plates were washed again, and then we applied 0–50 ng/mL homologous venom (*N. atra*) or heterologous venom (other venom of genus *Naja* from Asia or Africa and *O. hannah*), after which the plates were incubated for 1 h at 37 °C. The horse anti-*N. atra* F(ab’)_2_ (1.3 µg/mL in PBST) was then loaded and incubated, and a secondary antibody labeled with horseradish peroxidase was further bound for the chromogenic reaction. Between each step, the unbound antibodies were removed by the washing steps described above. The chromogenic reaction was initiated by adding TMB substrate and was stopped after 30 min of incubation by using 1 N HCl solution. The absorbance was measured at 450 nm by a Multiskan™ FC Microplate Photometer (Thermo Fisher Scientific, USA). All OD data were gained after background deduction, repeated for at least three times, and shown as mean ± SD.

### 5.4. Gel Electrophoresis and Coomassie Brilliant Blue Staining

The proteins from snake venom (3.3 µg) were separated by a 4%–12% Bis-Tris Plus Gel electrophoresis system. The samples were prepared under non-reducing conditions and electrophoresed at a constant voltage according to the manufacturer’s instructions. The protein molecular weight marker (Novex^®^ Sharp Pre-stained Protein Standard, 3.5–260 kDa) was loaded at the same volume as the samples in every gel. After electrophoresis, the gels were soaked in 0.1% Coomassie Brilliant Blue solution containing 50% methanol and 10% acetic acid and shaken for 1 h at room temperature. Subsequently, the staining solution was replaced by destaining buffer (40% methanol and 10% acetic acid) to remove the residual dye until the gel became transparent again. All experiments were repeated at least three times.

### 5.5. Western Blotting

After electrophoresis, the gels were placed in the electroblotting apparatus adjacent to a 0.22-µm PVDF membrane in buffer to transfer the separated proteins from the gel to the membrane. Then, the membranes were soaked with blocking buffer (5% skimmed milk in PBST), rabbit anti-*N. atra* IgG solution (0.55 µg/mL in PBST), and goat anti-rabbit-HRP (1:10,000 dilution) sequentially for one hour each. The goat anti-rabbit antibody was probed by using a chemiluminescent HRP substrate and detected by an ImageQuant LAS 4000 system (GE Healthcare, Chicago, IL, USA). All experiments were repeated at least three times.

### 5.6. Preparation of the Immunochromatographic Assay Kit (ICT-Cobra)

ICT-Cobra was manufactured by Formosa Biomedical Technology Corp., Taiwan and was assembled by following methods from a previous study with modifications [20]. The kit was prepared with nitrocellulose membranes, sample pads, conjugate pads, and absorbent pads that overlapped side-by-side. Briefly, the test lines were prepared with 1 mg/mL rabbit polyclonal anti-*N. atra* venom solution, and the control lines were prepared with 0.5 mg/mL goat anti-rabbit immunoglobulin antibody solution. Conjugation of colloidal gold and antibody was performed by incubating 2 µg of rabbit polyclonal anti-*N. atra* venom IgG and 1 mL of colloidal gold solution (O.D. of 3 at 540 nm) and gently stirring the resulting fluid. The test line, control line, and conjugate pad were all dispensed at a flow rate of 1 µL/cm and then dried at 37 °C for 4 h. All the components were assembled, cut into 0.4 mm-wide strips, and inserted into a plastic cassette. The ready-for-use devices were stored in sealed aluminum bags with desiccants.

### 5.7. Venom Detection with ICT-Cobra In Vitro

We had done a small-scale test and found no obvious difference in the detection limit between fresh human and fetal bovine serum (FBS). In order to avoid consumption of a large volume of precious human plasma, we chose the fetal bovine serum to perform these investigations. All venom samples were freshly dissolved in FBS at concentrations of 0, 5, 10, 50, 100, and 500 ng/mL, and 90 µL was loaded onto the sample zone of ICT-Cobra to test the detection limit. During the entire testing process, the kits must rest on a flat table, and it is necessary to avoid any shaking. The test was performed in triplicate and interpreted by the naked eye after 20 min by four laboratory staff members. Each test was interpreted 12 times for statistical analysis. The results are shown as the percentage (%) of positive interpretation. The lowest concentration resulting in 100% positive interpretation was defined as the detection limit.

## Figures and Tables

**Table 1:** The detection limits for ICT-Cobra on eight representative cobra venoms from Asia and Africa.

Venom	Venom Concentrations (ng/mL)					
	0	5	10	50	100	500
*N. atra*	0 ^a^	100	100	100	100	100
*N. siamensis*	0	75	100	100	100	100
*N. kaouthia*	0	100	100	100	100	100
*N. naja*	0	100	100	100	100	100
*N. nigricollis*	0	0	0	58.33	50	100
*N. haje*	0	16.7	25	100	100	100
*N. melanoleuca*	0	0	50	91.7	100	100
*O. hannah*	0	0	0	0	0	0

^a^ Numbers represents the percentage (%) of positive interpretation.

**Table 2:** The origin of the eight representative cobra venoms from Asia or Africa.

Venoms	Origin
*N. atra*	Taiwan
*N. kaouthia*	Thailand
*N. naja*	India
*N. siamensis*	Thailand
*N. nigricollis*	West Africa
*N. haje*	- ^a^
*N. melanoleuca*	Cameroon
*O. hannah*	Indonesia

^a^ No information can be obtained.

## Supplementary Materials

The following are available online at https://www.mdpi.com/2072-6651/12/9/572/s1, Figure S1: Reverse-phase high-performance liquid chromatography (RP-HPLC) profiles of the eight medically important snake venoms, Figure S2: Examples of the test of ICT-Cobra with venoms spiked in human serum in vitro.
