# Supplementary material for: A Rapid and International Applicable Diagnostic Device for Cobra (Genus Naja) Snakebites
Source: Toxins (Basel). 2020 Sep 5;12(9):572. doi: 10.3390/toxins12090572 (PMC7551368; doi:10.3390/toxins12090572)
Supplement: Supplementary file 1 [file toxins-12-00572-s001.pdf]

Supplementary Materials: A Rapid and International Applicable Diagnostic Device for Cobra (Genus *Naja*) Snakebites

Jing-Hua Lin, Wang-Chou Sung, Jiunn-Wang Liao and Dong-Zong Hung

Separation of Venom Proteins by Reverse-phase High-performance Liquid Chromatography (RP-HPLC)

Fractionation of eight venom components was performed with an HPLC system (Alliance 2695; Waters, Milford, MA, USA) equipped with a dual absorbance ultraviolet detector (Model 2487). The lyophilized venom dissolved in distilled water was subjected to reverse-phase column chromatography (250 × 4.6 mm, 5 μm particles with a 300 Å pore size; Jupiter C18, Phenomenex, Torrance, CA, USA) and eluted with a 0.8 mL/min flow rate with two mobile phases (mobile phase B: 0.1% trifluoroacetic acid (TFA); mobile phase C: 100% acetonitrile with the addition of 0.1% TFA); the gradient was as follows: 2% C for 5 min, 2–10% C for 2 min, 10–16% B for 6 min, 16–28% B for 2 min, 28–65% B for 37 min, 65–80% B for 3 min, and 2% C for 10 min. The absorbances (A215 and A280) of the eluate were monitored.

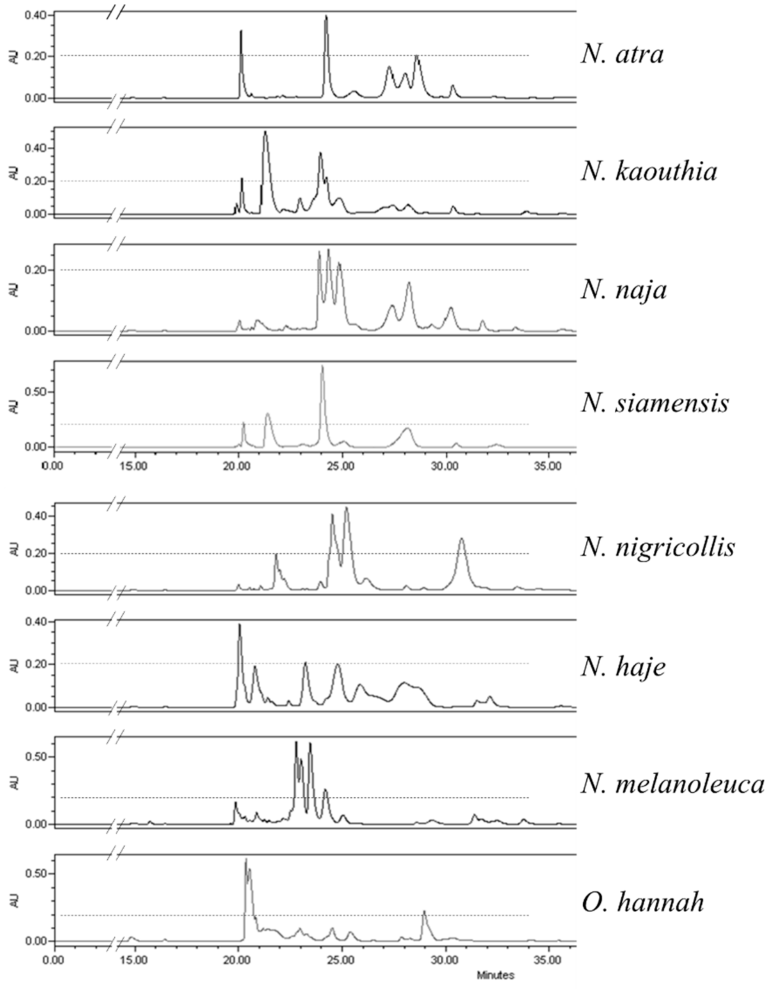

Figure S1. Reverse-phase high-performance liquid chromatography (RP-HPLC) profiles of the eight medically important snake venoms. The chromatograms showing the major peaks of venomous component eluted between 15 and 35 min were analyzed and aligned. For Asian cobras (*N. kaouthia*, *N. naja* and *N. siamensis*), at least three protein peaks appeared in the profiles, which is consistent with the peaks of *N. atra* venom. Except for *N. haje* venom, which produced two peaks consistent with those

of *N. atra* venom, the venoms of African cobras (*N. nigricollis* and *N. melanoleuca*) and *O. hannah* appeared only one peak or no peaks.

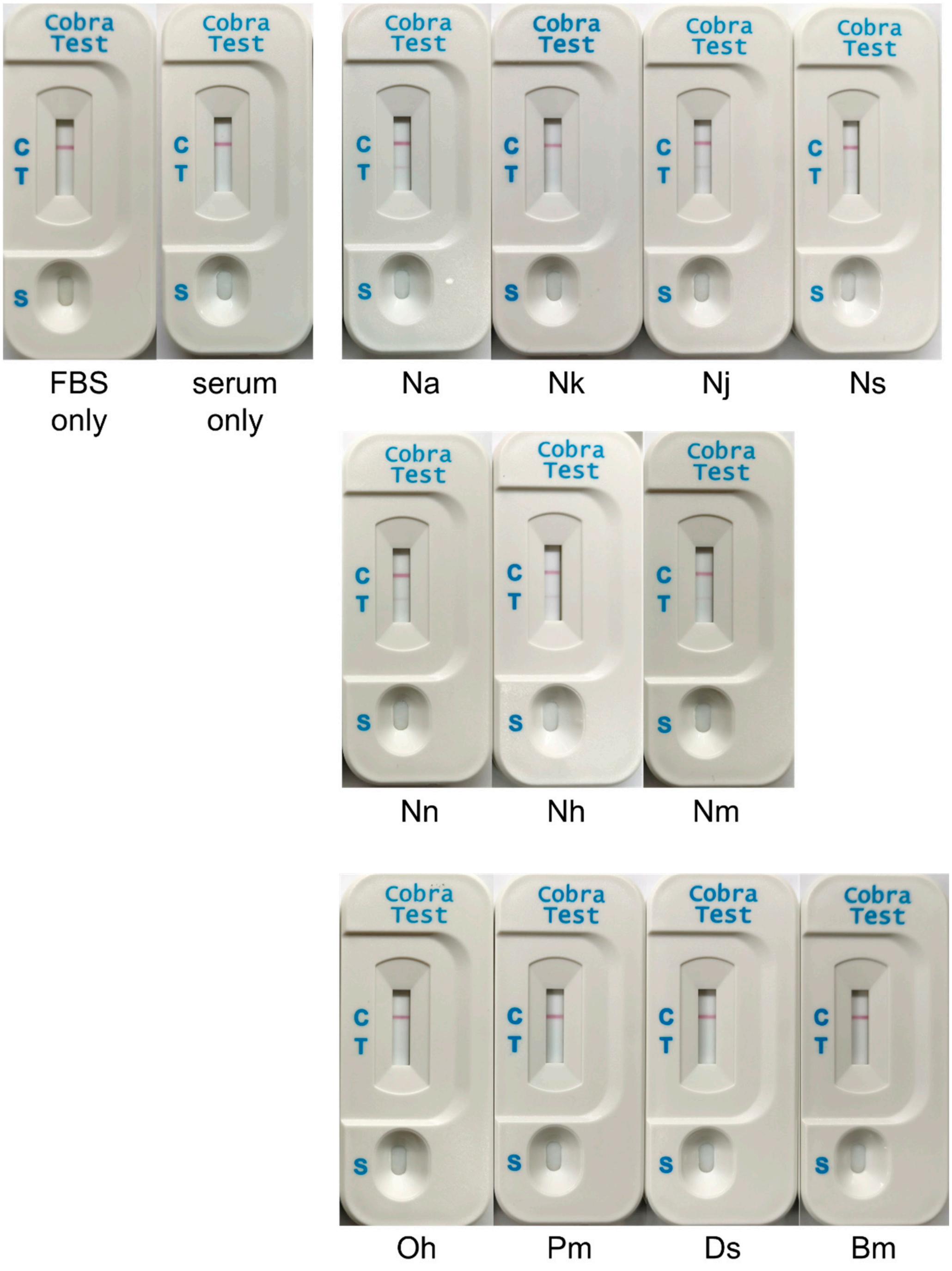

Figure S2. Examples of the test of ICT-Cobra with venoms spiked in human serum *in vitro*. Venom samples were dissolved in human serum at concentrations of 50 (Asian cobras) or 500 (African cobras, krait, and vipers) ng/mL. Total 90 µl of solution was loaded onto the sample zone of ICT-Cobra. These pictures were taken 20 min later. The labels on the plastic cassette: C, control line, checking the immunochromatographic function; T, test line, detecting the presence of venom in the sample; S, sample loading zone. Acronyms representing the venoms: *N. atra* (Na), *N. kaouthia* (Nk), *N. naja* (Nj), *N. siamensis* (Ns), *N. nigricollis* (Nn), *N. haje* (Nh), *N. melanoleuca* (Nm), *O. hannah* (Oh), *P. mucrosquamatus* (Pm), *D. siamensis* (Ds), and *B. multicinctus* (Bm).
